# Simvastatin, edaravone and dexamethasone protect against kainate-induced brain endothelial cell damage

**DOI:** 10.1186/s12987-019-0166-1

**Published:** 2020-02-10

**Authors:** Lilla Barna, Fruzsina R. Walter, András Harazin, Alexandra Bocsik, András Kincses, Vilmos Tubak, Katalin Jósvay, Ágnes Zvara, Patricia Campos-Bedolla, Mária A. Deli

**Affiliations:** 1 Institute of Biophysics, Biological Research Centre, Temesvári krt. 62, Szeged, 6726 Hungary; 2 Doctoral School in Biology, University of Szeged, Somogyi u. 4, Szeged, 6720 Hungary; 3 Creative Laboratory Ltd., Temesvári krt. 62, Szeged, 6726 Hungary; 4 Institute of Biochemistry, Biological Research Centre, Temesvári krt. 62, Szeged, 6726 Hungary; 5 Institute of Genetics, Biological Research Centre, Temesvári krt. 62, Szeged, 6726 Hungary; 6 Unidad de Investigacion Medica en Enfermedades Neurologicas, Hospital de Especialidades, Centro Medico Nacional Siglo XXI, Instituto Mexicano del Seguro Social, Av. Cuauhtémoc 330, Col. Doctores, 06720 Ciudad de México, DF México; 7 Department of Cell Biology and Molecular Medicine, University of Szeged, Szeged, Hungary

**Keywords:** Blood–brain barrier, Brain endothelial cells, Kainate, Simvastatin, Edaravone, Dexamethasone, Permeability, Reactive oxygen species, Nitric oxide synthase

## Abstract

**Background:**

Excitotoxicity is a central pathological pathway in many neurological diseases with blood–brain barrier (BBB) dysfunction. Kainate, an exogenous excitotoxin, induces epilepsy and BBB damage in animal models, but the direct effect of kainate on brain endothelial cells has not been studied in detail. Our aim was to examine the direct effects of kainate on cultured cells of the BBB and to test three anti-inflammatory and antioxidant drugs used in clinical practice, simvastatin, edaravone and dexamethasone, to protect against kainate-induced changes.

**Methods:**

Primary rat brain endothelial cell, pericyte and astroglia cultures were used to study cell viability by impedance measurement. BBB permeability was measured on a model made from the co-culture of the three cell types. The production of nitrogen monoxide and reactive oxygen species was followed by fluorescent probes. The mRNA expression of kainate receptors and nitric oxide synthases were studied by PCR.

**Results:**

Kainate damaged brain endothelial cells and made the immunostaining of junctional proteins claudin-5 and zonula occludens-1 discontinuous at the cell border indicating the opening of the barrier. The permeability of the BBB model for marker molecules fluorescein and albumin and the production of nitric oxide in brain endothelial cells were increased by kainate. Simvastatin, edaravone and dexamethasone protected against the reduced cell viability, increased permeability and the morphological changes in cellular junctions caused by kainate. Dexamethasone attenuated the elevated nitric oxide production and decreased the inducible nitric oxide synthase (NOS2/iNOS) mRNA expression increased by kainate treatment.

**Conclusion:**

Kainate directly damaged cultured brain endothelial cells. Simvastatin, edaravone and dexamethasone protected the BBB model against kainate-induced changes. Our results confirmed the potential clinical usefulness of these drugs to attenuate BBB damage.

## Introduction

Excitotoxicity has a pivotal role in many neurological diseases, including stroke, traumatic brain injury, epilepsy and neurodegenerative disorders like multiple sclerosis, Alzheimer’s, Huntington’s and Parkinson’s diseases [1–3]. Glutamate is one of the most important excitatory neurotransmitters of the CNS, and together with endogenous or exogenous excitotoxins, like *N*-methyl-d-aspartate (NMDA), α-amino-3-hydroxy-5-methyl-4-isoxazolepropionic acid (AMPA) and kainate, act on specific receptor families. Receptors of glutamate (NMDA receptors: GluN1-3B; AMPA receptors: GluA1-4; kainate receptors: GluK1-5; metabotropic receptors: mGluR1-8) are highly expressed in different brain areas such as cortex, limbic system, basal ganglions, hippocampus and cerebellum [4]. Excitatory neurotransmitters are fundamental for physiological processes, but the excessive stimulation of these receptors causes excitotoxicity, the damage or death of the nerve cells [4]. Kainate is a natural glutamate analogue isolated from seaweed which can bind to glutamate receptors. In research kainate is used to induce epilepsy in animal experiments in which not only excitotoxicity and neuronal damage but also blood–brain barrier (BBB) leakage and neurovascular changes are observed [5]. Among the excitatory compounds the effect of glutamate and the presence of glutamate receptors on brain endothelial cells have been described previously by our group and others [6–11], but kainate effects and receptors are less investigated at the level of BBB.

Taking into account the central role of the BBB in central nervous system (CNS) physiology [12] and neuropathologies [13] the cerebral vasculature emerges as a therapeutic target for neurological diseases [14, 15]. Vascular inflammation and oxidative stress are central pathways in many CNS diseases such as stroke, amyotrophic lateral sclerosis and epilepsy, and anti-inflammatory or antioxidant drugs are also used to treat them [15–17]. For the present study we selected three clinically used drugs, the anti-inflammatory simvastatin and dexamethasone, and the free radical scavenger edaravone. Besides their lipid-lowering effect, statins also exhibit neuroprotective, immunosuppressive, anticonvulsant and antioxidant properties [18, 19]. The pleiotropic effects of statins include the inhibition of inflammatory responses and the improvement of endothelial functions [20]. Simvastatin is a lipophilic statin exerting neuroprotective effects [21], which also protects the BBB in an acute stroke model in rats [22]. Edaravone is an excellent free radical scavenger molecule, which is clinically used for treating acute stroke and amyotrophic lateral sclerosis [17]. Our group demonstrated the protective effect of edaravone on brain endothelial cells against methylglyoxal-induced barrier damage [23]. In a kainate-induced epilepsy model in rats edaravone significantly decreased neuronal cell death and hyperexcitability [24]. Dexamethasone, a synthetic corticosteroid, has a strong anti-inflammatory and immunosuppressant effects. It also enhances barrier properties in culture models of the BBB, including elevation of transendothelial electrical resistance, decrease in paracellular permeability and upregulation of tight junction proteins [25, 26]. Dexamethasone was protective in animal models, too: it decreased the BBB permeability and edema in kainate-induced seizures in rats [27] and protected the BBB from damage and reduced the severity of seizures in pilocarpin-induced status epilepticus [16]. In addition, dexamethasone exerted beneficial effects in pediatric drug resistant epileptic patients [16].

Our aim was to investigate the direct effect of kainate on culture models of the three major cell types of the BBB, brain endothelial cells, pericytes and astrocytes, and to test clinically used therapeutic molecules simvastatin, edaravone and dexamethasone as potential protective agents against kainate-induced brain endothelial damage using a BBB co-culture model.

## Materials and methods

### Materials

All reagents were purchased from Sigma-Aldrich Corporation (subsidiary of Merck, Darmstadt, Germany), unless otherwise indicated. The source and catalogue number of the reagents are listed in Additional file 1: Table S1.

### Cell cultures

The isolation of primary rat brain endothelial cells, glial cells and pericytes and the co-culture BBB model were done as described in our previous studies [28, 29] and in Additional file 1: Detailed protocol. Primary rat brain endothelial cells were isolated from 3-week-old outbred male and female Wistar rats (Harlan Laboratories, USA). Following isolation cells were seeded onto collagen type IV and fibronectin coated (100 µg/ml each) Petri dishes (100 mm; Corning, USA). Cells were maintained in DMEM-F12 supplemented with 15% plasma-derived bovine serum (PDS; First Link, UK), 5 μg/ml insulin, 5 μg/ml transferrin, 5 ng/ml sodium selenite (Pan Biotech, Germany), 10 mM Hepes, 1 ng/ml basic fibroblast growth factor, 100 μg/ml heparin and 50 μg/ml gentamycin. During the first 3 days of culture the capillary endothelial cells were kept in culture medium containing 3 μg/ml puromycin to eliminate P-glycoprotein negative cell types [30]. Cells were used at the first passage (P1) for experiments.

Primary rat brain pericytes were isolated by the same protocol, except that puromycin treatment was not applied. After isolation pericytes were seeded onto collagen type IV coated (100 µg/ml) Petri dishes (60 mm; VWR International, USA) and cultured in DMEM medium (Gibco, Life Technologies, USA) containing 10% fetal bovine serum (FBS, Pan Biotech, Germany) and gentamycin (50 μg/ml). Cells were used at the third passage (P3) for experiments.

Primary rat astrocytes were obtained from 1-day-old Wistar rats. Following mechanical dissociation of brain tissue and filtration, cell clusters were plated onto uncoated 75 cm^2^ flasks (TPP, Switzerland) and cultured in DMEM supplemented with 10% FBS and gentamycin (50 μg/ml) until they reached 90% confluency. Cells were passaged (P1) to collagen type IV (100 µg/ml) coated 12-well plates (Corning, USA) at a cell number of 5 × 10^4^ cells/well and cultured for 2 weeks before use in the co-culture model. Glia cultures included more than 90% GFAP immunopositive astrocytes (Additional file 1: Figure S1).

For the co-culture BBB model [31] first pericytes (1.5 × 10^4^ cells/cm^2^) were passaged to the bottom side of the 12-well format culture inserts (Transwell clear, polyester membrane, 0.4 µm pore size, 1.12 cm^2^ surface; Corning, USA) coated with collagen type IV (100 µg/ml), then brain endothelial cells (7.5 × 10^4^ cells/cm^2^) were added to the upper side coated with collagen type IV and fibronectin (100 µg/ml each). The inserts were placed into 12-well plates containing confluent P1 astrocyte layers. Endothelial culture medium was added to both compartments. Cells were co-cultured for 4 days before experiments [30, 31]. For longer version of the cell culture protocol and immunostaining of pericytes (see Additional file 1: Detailed protocol, Figure S1).

### Treatments

Rat brain endothelial cells, astrocytes and pericytes were treated with kainate (213 Da; 100 mM stock solution was prepared in sterile water by the addition of NaOH) at 10 µM and 100 µM concentrations in culture medium for 1 and 24 h, based on our preliminary experiment and in agreement with literature data [32]. Simvastatin, edaravone (for both 1 mM stock solution was prepared in dimethyl sulfoxide) and dexamethasone (cyclodextrin complex; 10 mM stock solution was prepared in sterile water) were applied at a concentration of 1 µM based on our preliminary study and literature data [21, 23, 25]. The control group received culture medium. Triton X-100 (TX-100) detergent was used at 1% concentration in viability assays as a reference compound to cause cell death (Additional file 1: Figure S2). As reference compounds hydrogen peroxide (H_2_O_2_; 100 µM) was used for the measurement of reactive oxygen species and sodium nitroprusside (SNP; 100 µM) during the nitric oxide production assay.

### Real-time cell analysis

For dynamic monitoring of living brain endothelial cells impedance-based cell electronic sensing was used, which is a non-invasive, label-free technique. This method has been established to follow not only cell attachment and growth, but cell viability as well. We have verified by endpoint colorimetric tests and morphological methods that impedance changes can sensitively detect cell damage [23, 29, 33] and protection [23, 29]. The RTCA-SP system (ACEA Biosciences, CA, USA) registers the impedance of cells automatically every 10 min. For every time point cell index is defined as (R_n − _R_b_)/15, where R_n_ is the impedance of the wells containing cells and R_b_ means the background impedance of the wells containing medium but not cells. Cells were passaged to special 96-well microtiter plates with gold electrodes (E-plate, ACEA Biosciences, CA, USA) which were coated with collagen type IV and fibronectin (100 µg/ml each) for rat brain endothelial cells or with collagen type I (150 µg/ml) for rat astrocytes and pericytes. For measuring background impedance 50 µl culture medium was added to each well, then 50 µl cell suspension was seeded at a density of 6 × 10^3^ cells/well. When the impedance of cells reached a plateau phase, they were treated with kainate and the selected drugs and were monitored for an additional 24 h. As a reference compound to induce cell death Triton X-100 (TX-100) detergent was used at 1% concentration (Additional file 1: Figure S2).

### Total RNA isolation and reverse transcription polymerase chain reaction (RT-PCR)

Total RNA was isolated from rat brain cortex, microvessels and brain endothelial cells by using TriFast reagent (VWR International, USA), then 1 µg RNA from each sample was transcribed to complementary DNA by Maxima First Strand cDNA Synthesis Kit (Thermo Fisher, USA). Previously designed specific oligonucleotide primer pairs, covering different exons or exon/exon boundaries, were used and modified for the five rat kainate receptor genes [34–36] and for the three nitric oxide synthase genes [37] (Additional file 1: Table S2). Primers for β-actin and glyceraldehyde 3-phosphate dehydrogenase (GAPDH) genes were used as loading controls (Additional file 1: Table S2). PCR was performed with FIREPol DNA Polymerase (Solis BioDyne, Estonia) in Labcycler 48 s Gradient (SensoQuest, Germany). After heat inactivation for 3 min at 95 °C, the cycling conditions were the following: denaturation for 30 s at 95 °C, annealing for 30 s at the appropriate annealing temperature (Additional file 1: Table S2), polymerization for 40 s at 72 °C (35 cycles), final extension for 5 min at 72 °C. Products were analysed on 2% agarose gel (VWR International, USA), then isolated fragments were sequence verified by capillary DNA sequencing. Band intensities on the gel photos were quantified by ImageJ software (National Institutes of Health, USA). The qPCR was performed with 2 × Power SYBR Green PCR Master Mix (Applied Biosystems, USA) in a RotorGene 3000 instrument (Corbett Research, Australia). After heat activation at 95 °C for 2 min the cycling conditions were the following: denaturation for 5 s at 95 °C, annealing and polymerization for 30 s at 60 °C (40 cycles). Fluorescent signals were collected after each extension step and at the end the registration of the melting curve was performed between 55 and 95 °C. Relative gene expression levels were normalized to endogenous control genes (β-actin and GAPDH) (ΔCt). Then ΔΔCt was calculated in comparison to the relative expression of the target genes in untreated control groups. Fold changes were calculated using the 2^−ΔΔCt^ formula.

### Measurement of permeability for marker molecules

Two marker molecules were used for the permeability measurement: Evans blue-labeled albumin (EBA: 1% BSA + 167.5 µg/ml Evans blue; 67 kDa) and sodium fluorescein (SF; 10 µg/ml; 376 Da). After treatment, inserts were placed into 12-well plates containing 1.5 ml Ringer–Hepes buffer (150 mM NaCl, 2.2 mM CaCl_2_, 0.2 mM MgCl_2_, 5.2 mM KCl, 5 mM glucose, 6 mM NaHCO_3_ and 10 mM Hepes; pH 7.4) supplemented with insulin, transferrin and sodium selenite and 0.1% BSA. The culture medium was changed in the upper compartment to 0.5 ml Ringer–Hepes buffer containing EBA and SF. For 30 min the plates were kept on a horizontal shaker (150 rpm) in a CO_2_ incubator then samples were collected from both compartments. The concentrations of the marker molecules were measured by a fluorescence multiwell plate reader (Fluostar Optima, BMG Labtechnologies, Germany) at 584 nm excitation and 680 nm emission wavelengths for EBA, and 485 nm excitation and 520 nm emission wavelengths for SF. The apparent permeability coefficients (P_app_) were calculated as described in our earlier studies [29, 38].

### Measurement of reactive oxygen species and nitric oxide production

Chloromethyl-dichloro-dihydro-fluorescein diacetate (DCFDA, Molecular Probes, Life Technologies, USA) was used for ROS detection, and 4-amino-5-methylamino-2′,7′-difluorofluorescein diacetate (DAF-FM, Molecular Probes, Life Technologies, USA) for NO detection as fluorescent probes, as described in our previous studies [39, 40]. Brain endothelial cells were cultured in 96-well plates with black walls and transparent plastic bottoms (Corning, NY, USA). After treatments for 1 or 24 h, cells were incubated in Ringer–Hepes buffer containing 2 µM DCFDA or 2 µM DAF-FM probes. Pluronic acid (Molecular Probes, Life Technologies, USA; 16 µM) was used to help the probes crossing the cell membrane. Fluorescence was detected by Fluostar Optima multiwell plate reader (BMG Labtechnologies, Germany) at 485 nm excitation and 538 nm emission wavelengths at every 3 min for 1 h.

### Immunohistochemistry

For the immunostaining of junctional proteins claudin-5 and zonula occludens-1 (ZO-1) rat brain endothelial cells (2.5 × 10^4^ cells/well) were grown on glass coverslips (1 cm^2^, borosilicate, VWR, USA) coated overnight at 4 °C with rat tail collagen (150 µg/ml). Cells treated with kainate and protective drugs for 24 h were fixed with 1% paraformaldehyde for 20 min at 4 °C, permeabilized with 0.2% Triton-X100 for 10 min at 4 °C, then blocked with 3% BSA-PBS for 1 h at room temperature. Samples were incubated with rabbit anti-claudin-5 polyclonal (SAB4502981, 1:800 dilution in 3% BSA-PBS, antibody registry ID: AB_10753223; Sigma-Aldrich, USA) or rabbit anti-ZO-1 polyclonal (61-7300, 1:400 dilution in 3% BSA-PBS, antibody registry ID: AB_2533147; Invitrogen, USA) primary antibodies overnight at 4 °C, then with anti-rabbit secondary antibody conjugated with Cy3 (C2306, 1:400 dilution in PBS, antibody registry ID: AB_258792; Sigma-Aldrich, USA) and H33342 nucleus stain (1 µg/ml) for 1 h at room temperature [40]. Cells were washed with PBS between the incubations. Coverslips were mounted in Fluoromount-G (Southern Biotech, USA) and visualized by Leica SP5 confocal laser scanning microscope. Tight junction staining patterns (4–7 images in each group) were analysed by MATLAB software (MathWorks, Natick, MA, USA). The backgrounds of each image were determined and subtracted to compensate the occasional non-uniform background. The grayscale images were converted to binary. The object number reveals the number of the separated structure elements of the images, indicating discontinuity in the staining pattern.

### Statistical analysis

Data are presented as mean ± SD or SEM. Intensity measurements for nitric oxide synthase (NOS) RT-PCR were analysed by ImageJ software (National Institutes of Health, USA). Statistical analysis was done by GraphPad Prism 5.0 software (GraphPad Software, USA). Significance between groups was determined using t-test or one-way ANOVA followed by Dunnett or Bonferroni posttests. Differences were considered statistically significant at *p* < 0.05. All experiments were repeated at least twice.

## Results

### Effect of kainate on cell viability of rat brain endothelial cells, astrocytes and pericytes

**Fig. 1:**
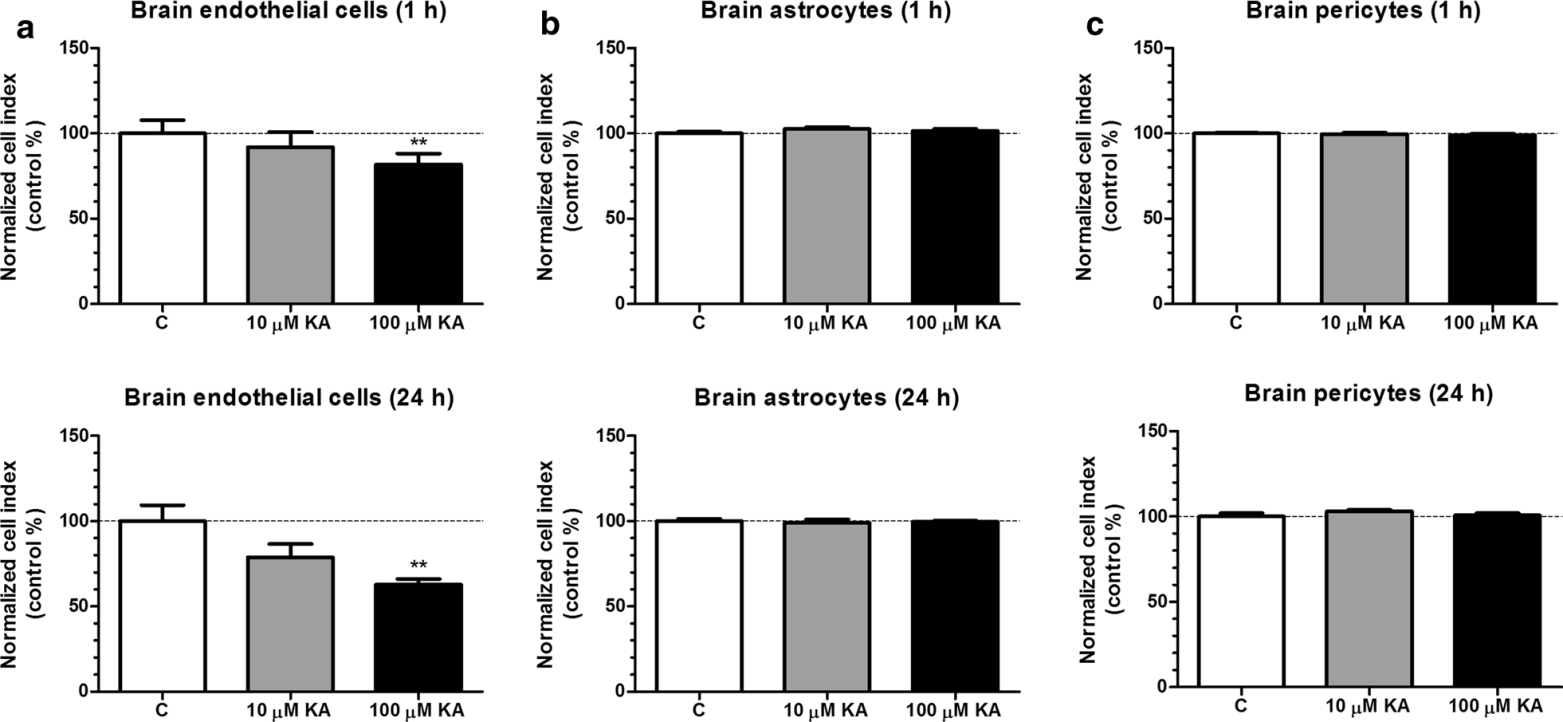
The effect of kainate on cell viability. **a** Cultured rat brain endothelial cells. **b** Rat astrocyte cultures. **c** Rat brain pericyte cultures after 1 h or 24 h treatment with kainate (KA). Control group (C) received only culture medium. Mean ± SEM, n = 3–12, ANOVA, Bonferroni-test, **p < 0.01 compared to the control group

Viability and integrity of brain endothelial, astrocyte and pericyte cell layers were monitored with real-time impedance measurement for 24 h after kainate treatment (10 and 100 μM) (Fig. 1a). Kainate at 100 μM concentration decreased endothelial cell viability compared to the control at both time points. Interestingly, kainate had no effect on cultured astrocytes and pericytes (Fig. 1b, c). Based on this result the 100 μM kainate treatment was selected for further experiments.

### Expression of kainate receptors in rat brain cortex, rat brain microvessels and primary rat brain endothelial cells

**Fig. 2:**
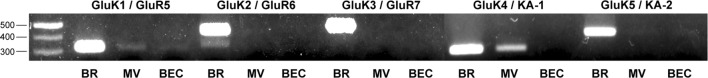
Expression of kainate receptors. GluK1/GluR5, GluK2/GluR6, GluK3/GluR7, GluK4/KA-1 and GluK5/KA-2 mRNA expression is shown in rat brain cortex (BR), isolated rat brain microvessels (MV) and primary rat brain endothelial cells (BEC). The predicted length of the products is shown in Additional file 1: Table S2. Fragments were visualized on 2% agarose gel. M: 1 Kb Plus DNA ladder

In rat brain cortex all of the five kainate receptor genes were expressed (GluK1/GluR5, GluK2/GluR6, GluK3/GluR7, GluK4/KA-1 and GluK5/KA-2) as shown in Fig. 2. In isolated rat brain microvessels the expression of GluK1 and GluK4, while in primary rat brain endothelial cell cultures GluK1 expression was detected by RT-PCR. GluK5 gene expression was also slightly detectable in these samples (Fig. 2). These results were verified by capillary DNA sequencing. The expression of housekeeping genes β-actin and GAPDH were similar in rat brain cortex, isolated rat brain microvessel and primary rat brain endothelial cell samples (Additional file 1: Figure S3).

### Effect of kainate on the permeability of the BBB co-culture model

**Fig. 3:**
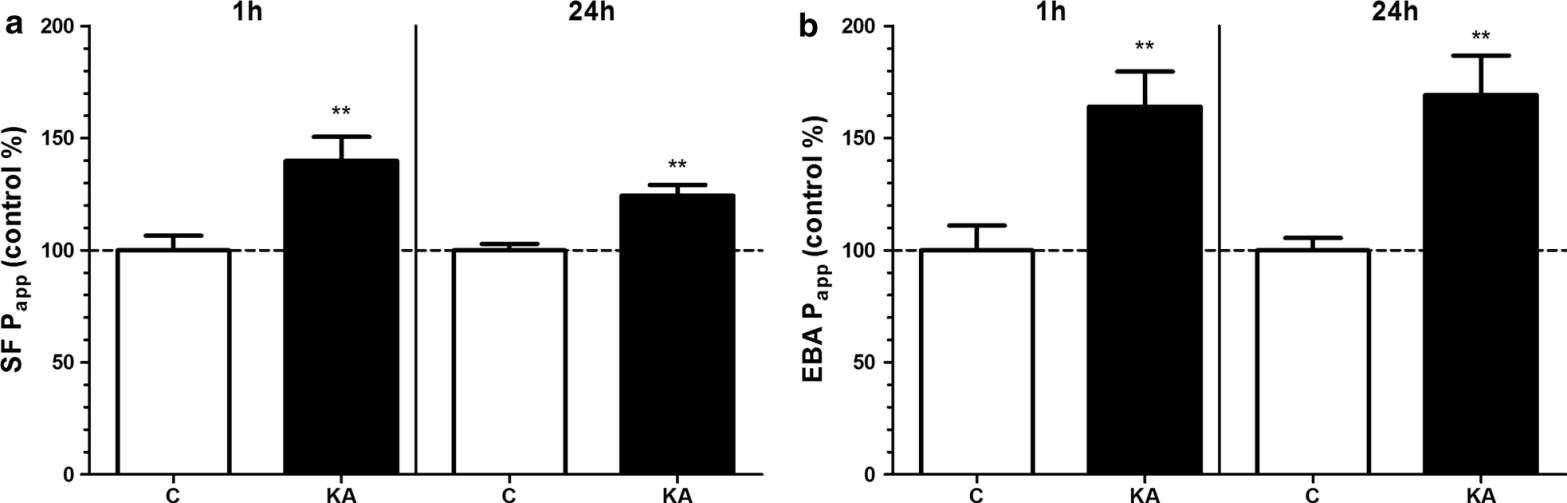
The effect of kainate on the permeability of the BBB co-culture model. **a** Permeability for sodium fluorescein (SF; 376 Da). **b** Permeability for Evans blue-labelled albumin (EBA; 67 kDa). Treatment with 100 µM kainate (KA) lasted for 1 or 24 h. Control groups (C) received only culture medium. Mean ± SD, n = 4–8, *t*-test, *p < 0.05, ***p < 0.001 compared to the control group. Permeability is expressed as apparent permeability coefficient (P_app_)

The tightness of the BBB model was tested by the measurement of P_app_ for marker molecules fluorescein (4.72 × 10^−6^ cm/s) and albumin (0.31 × 10^−6^ cm/s) which were in the range we measured in previous studies [29, 41]. Kainate treatment (100 μM) significantly increased the permeability of the BBB model for both markers at the 1 and 24 h time points (Fig. 3).

### Effect of kainate on reactive oxygen species and nitric oxide production

**Fig. 4:**
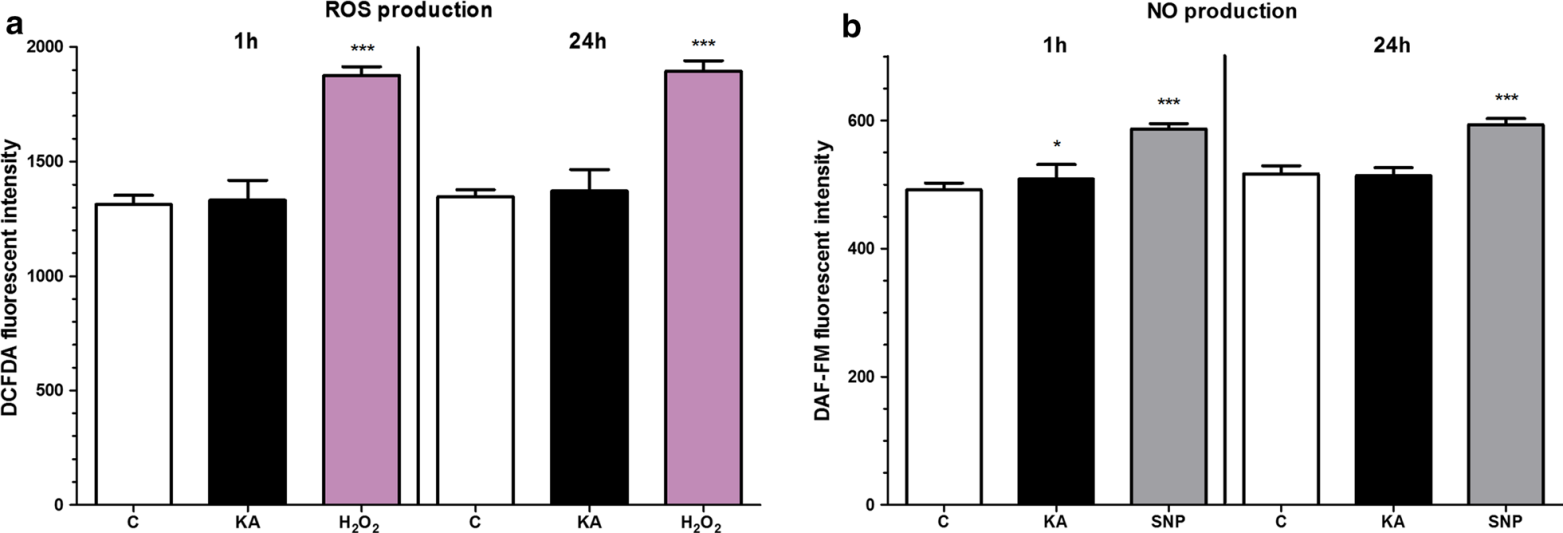
The effect of kainate treatment on primary rat brain endothelial cells. **a** Reactive oxygen species (ROS) production. **b** Nitric oxide (NO) production. Cells were treated with 100 µM kainate (KA) for 1 or 24 h. Control groups (C) received only culture medium. Hydrogen peroxide (H_2_O_2,_ 100 µM) served as reference compound in the ROS measurement, and sodium nitroprusside (SNP, 100 µM) served as reference compound in the NO assay. Mean ± SD, n = 4–6, ANOVA and Dunnett test, *p < 0.05, ***p < 0.001 compared to the control group

Kainate did not show any effect on the basal ROS production of brain endothelial cells after 1 or 24 h compared to the control group, but it elevated NO generation in brain endothelial cells after 1 h (Fig. 4). Hydrogen peroxide was used as a reference inducer of ROS in the assay (Fig. 4), as in our previous study [29], while sodium nitroprusside was given to increase NO production [40].

### Effect of simvastatin, edaravone and dexamethasone on the cell viability of kainate-treated brain endothelial cells

**Fig. 5:**
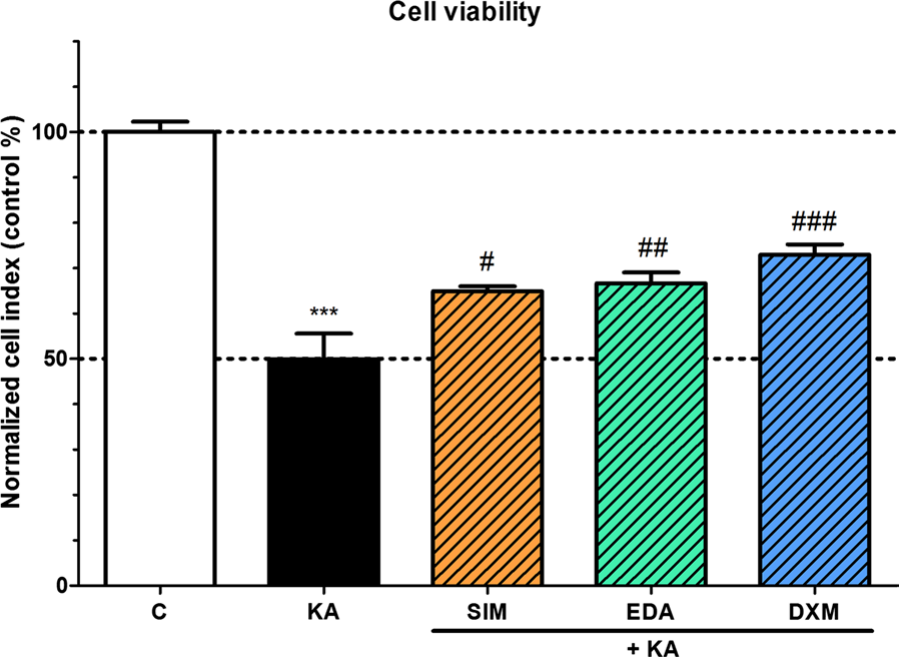
The effect of kainate and protective compounds on cell viability of primary rat brain endothelial cells. Rat brain endothelial cells were treated with 100 µM kainate (KA) without or with simvastatin (SIM, 1 µM), edaravone (EDA, 1 µM) or dexamethasone (DXM, 1 µM) for 24 h. Control groups (C) received only culture medium. Mean ± SD, n = 4–8, ANOVA, Bonferroni test, ***p < 0.001 compared to the control group, ^#^*p* < 0.05, ^##^*p* < 0.01, ^###^*p* < 0.001 compared to the kainate-treated group

Simvastatin, edaravone or dexamethasone at 1 μM concentration significantly increased cell viability measured by the impedance assay and compared to the kainate treatment group (100 μM) as reflected by the elevated cell index of rat brain endothelial cells at 24 h timepoint (Fig. 5).

### Effect of simvastatin, edaravone and dexamethasone on the permeability of kainate-treated BBB co-culture model

**Fig. 6:**
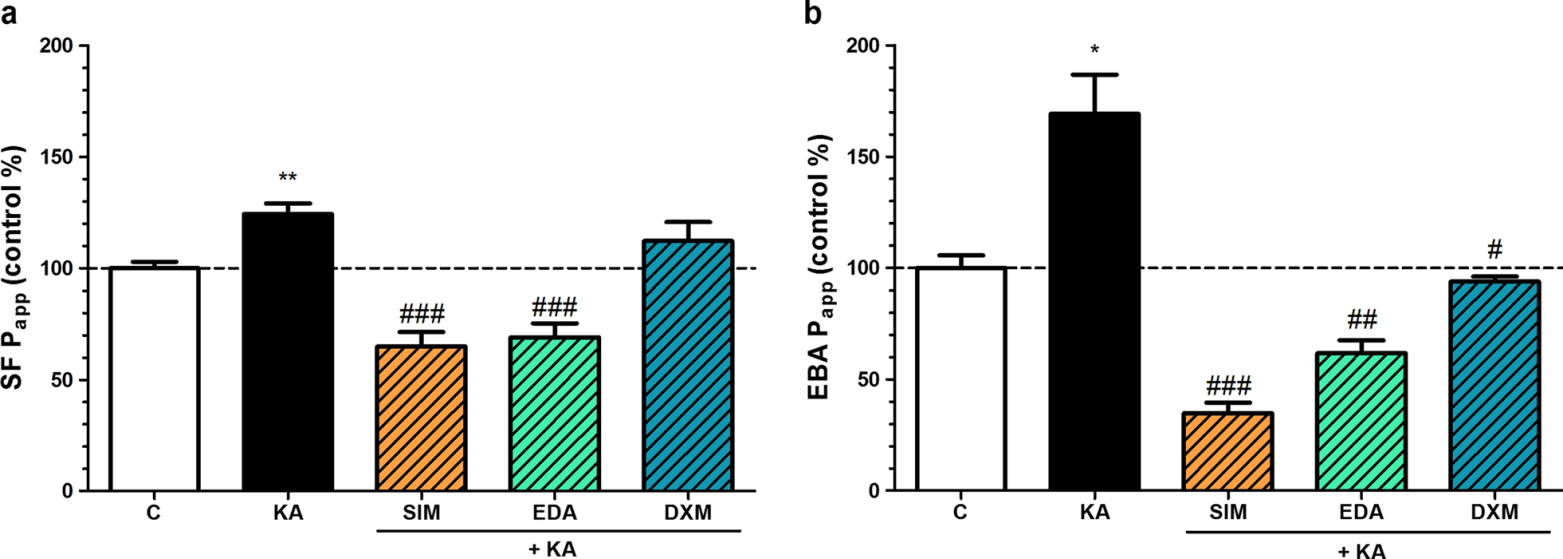
The effect of kainate and protective compounds on the permeability of BBB co-culture model. **a** Permeability for sodium fluorescein (SF; 376 Da). **b** Permeability for Evans blue-labelled albumin (EBA; 67 kDa). Primary brain endothelial cells co-cultured with astrocytes and pericytes on inserts were treated with 100 µM kainate (KA) without or with simvastatin (SIM, 1 µM), edaravone (EDA, 1 µM) or dexamethasone (DXM, 1 µM) for 24 h. Control groups (C) received only culture medium. Mean ± SEM, n = 3–9, ANOVA, Bonferroni-test, **p < 0.01 compared to the control; ^#^*p* < 0.05, ^###^*p* < 0.001 compared to the kainate- treated group. Permeability is expressed as apparent permeability coefficient (P_app_) in the  % of the control values

To test the protective molecules, the permeability measurements were performed similarly as described above. In this assay simvastatin and edaravone significantly attenuated the increased permeability for both markers caused by kainate treatment, while dexamethasone blocked the barrier opening effect only for the transcellular marker albumin (Fig. 6).

### Effect of simvastatin, edaravone and dexamethasone on the morphology of kainate-treated endothelial cells

**Fig. 7:**
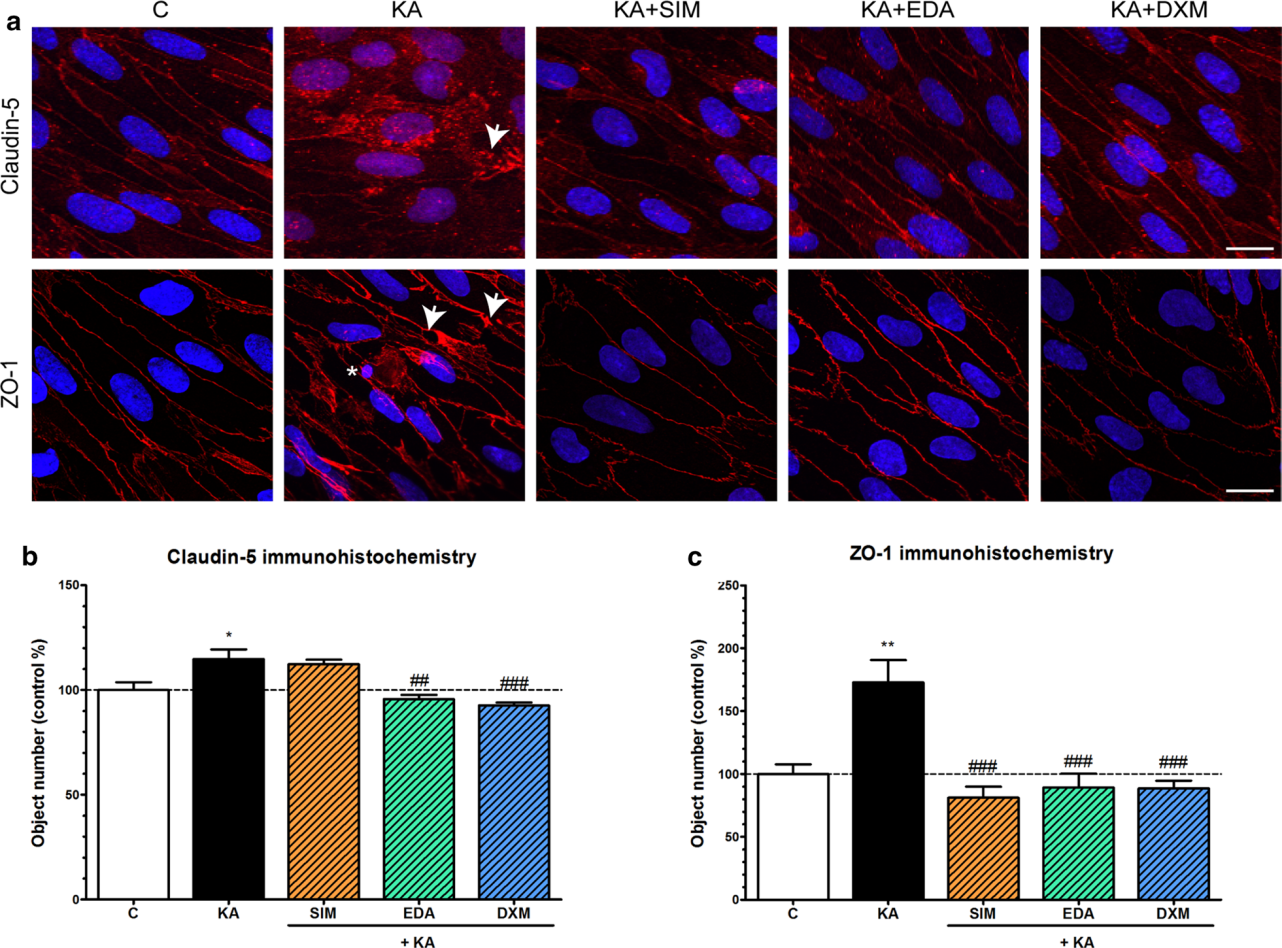
The effect of kainate treatment on the immunostaining of tight junction proteins claudin-5 and zonula occludens-1 in primary rat brain endothelial cells. **a** Representative confocal microscopy images of the immunostaining of junctional proteins claudin-5 and zonula occludens-1 (ZO-1) in rat brain endothelial cells treated with 100 µM kainate (KA) alone or with simvastatin (KA + SIM, 1 µM), edaravone (KA + EDA, 1 µM) or dexamethasone (KA + DXM, 1 µM) for 24 h. Control groups (C) received only culture medium. Red: junctional protein staining, blue: cell nuclei. Arrowheads: fragmented junctional staining and gaps, asterisks: apoptotic body. n = 6–10. Scale bar: 10 µm. **b** The object number on the claudin-5 immunostained pictures was quantified by MATLAB software. **c** The object number on the ZO-1 immunostained pictures was quantified by MATLAB software. Mean ± SEM, n = 4–7, ANOVA, Bonferroni test, *p < 0.05, **p < 0.01 compared to the control group, ^##^*p* < 0.01, ^###^*p* < 0.001 compared to the kainate-treated group

The tight paracellular barrier formed by brain endothelial cells in the BBB model was visualized by the continuous, belt-like pericellular localization of integral membrane tight junction protein claudin-5 and linker protein ZO-1 (Fig. 7a). In the kainate treated group junctional staining patterns of claudin-5 and ZO-1 were weaker and more fragmented at the cell border, as compared to the control group, while in cells treated with simvastatin, edaravone or dexamethasone the staining patterns of tight junction proteins were more similar to the control group (Fig. 7a). The object number on the immunostained pictures significantly increased due to kainate treatment as compared to the control group indicating a disturbance in the proper localization of the junctional proteins. Edaravone and dexamethasone significantly decreased the object number compared to the kainate-treated group for both immunostainings, while simvastatin was efficient to reduce the discontinuous pattern of ZO-1 immunostaining (Fig. 7b, c).

### Effect of simvastatin, edaravone and dexamethasone on reactive oxygen species and nitric oxide production of kainate-treated brain endothelial cells

**Fig. 8:**
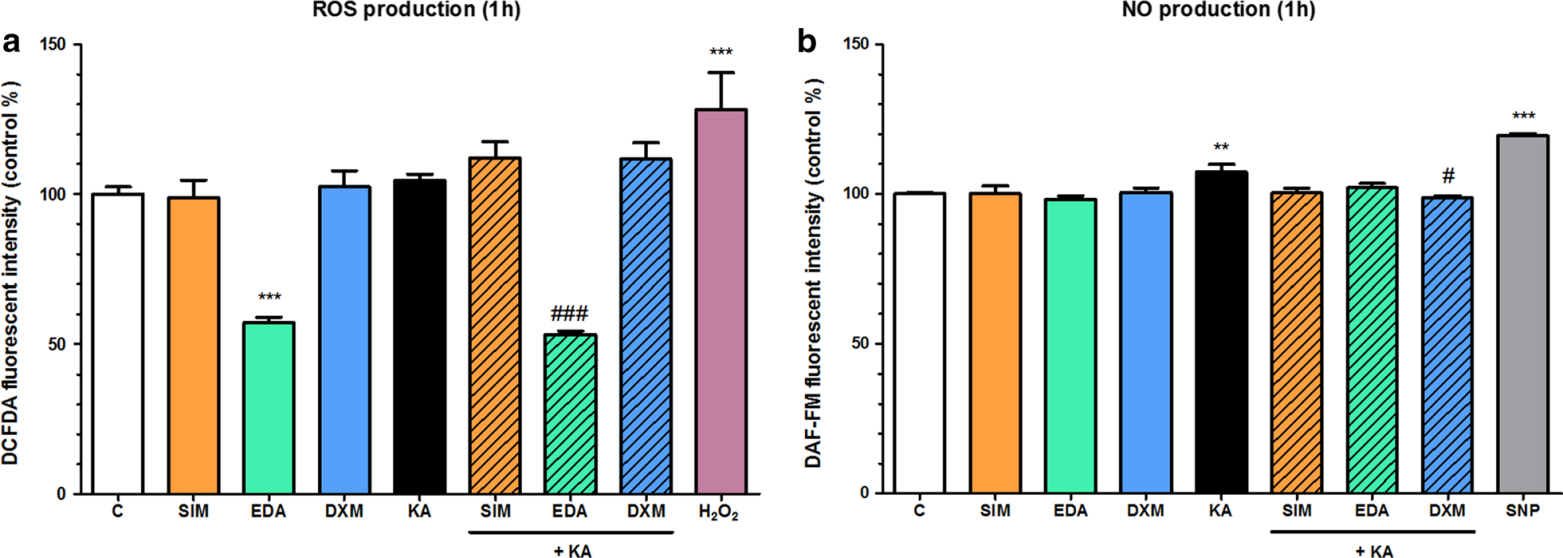
The effect of kainate treatment on primary rat brain endothelial cells. **a** Reactive oxygen species (ROS) production. **b** Nitric oxide (NO) production. Cells were treated with 100 µM kainate (KA) without or with simvastatin (SIM, 1 µM), edaravone (EDA, 1 µM) or dexamethasone (DXM, 1 µM) for 1 h. Control groups (C) received only culture medium. Hydrogen peroxide (H_2_O_2_, 100 µM) served as reference compound in the ROS measurement, and sodium nitroprusside (SNP, 100 µM) was used to release NO. Mean ± SD, n = 5–13, ANOVA, Bonferroni test, **p < 0.01, ***p < 0.001 compared to the control group, ^#^*p* < 0.05, ^###^*p* < 0.001 compared to the kainate-treated group

Although kainate did not influence the ROS production of brain endothelial cells we tested the effect of simvastatin, edaravone and dexamethasone in this experiment. Here edaravone showed a strong antioxidant effect on the basal ROS production alone or when it was given together with kainate (Fig. 8a). Simvastatin and dexamethasone had no effect on the generation of ROS in brain endothelial cells. More importantly, dexamethasone significantly reduced the elevated NO production induced by short-term kainate treatment (Fig. 8b).

### Expression of nitric oxide synthase genes in brain endothelial cells

**Fig. 9:**
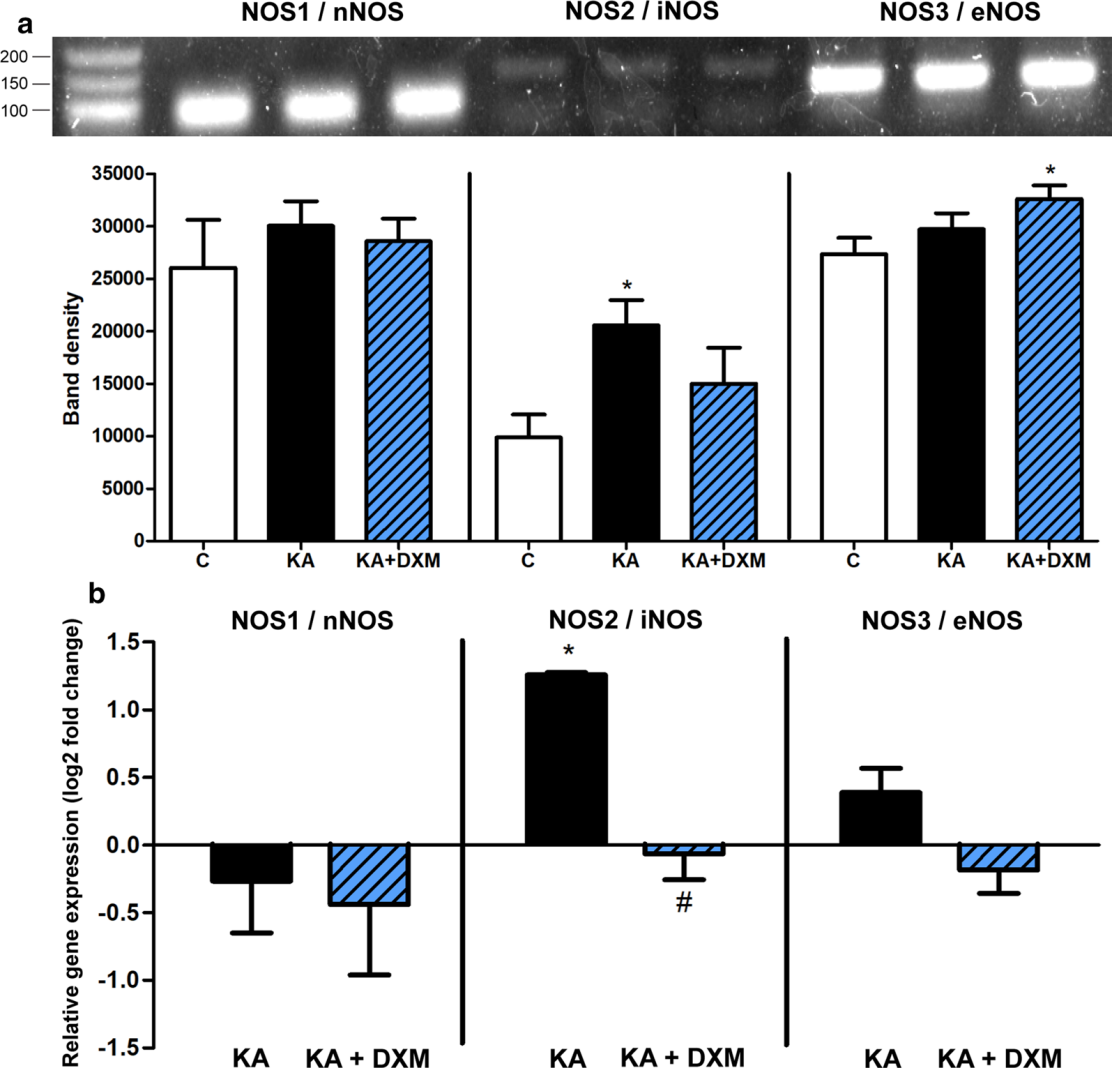
Expression of nitric oxide synthases in primary rat brain endothelial cells. **a** Expression of neuronal nitric oxide synthase (nNOS/NOS1), inducible nitric oxide synthase (iNOS/NOS2) and endothelial nitric oxide synthase (eNOS/NOS3) mRNA in primary rat brain endothelial cells treated with 100 µM kainate (KA) alone and with dexamethasone (KA + DXM, 1 µM) for 24 h. Control groups (C) received only culture medium. The predicted length of the products is shown in Additional file 1: Table S2. Fragments were visualized on 2% agarose gel. M: 1 Kb Plus DNA ladder. **b** Relative gene expression changes of nitric oxide synthases measured by qPCR. Fold change is the expression ratio compared to untreated control groups, where negative values mean target gene downregulation, positive values mean target gene upregulation. Mean ± SEM, n = 4, ANOVA, Bonferroni test, *p < 0.05 compared to the control group; ^#^*p* < 0.05 compared to the kainate-treated group

The expression of all three nitric oxide synthase genes was detected in rat brain endothelial cell cultures with RT-PCR and qPCR (Fig. 9). The endothelial NOS3 and the neuronal NOS1 genes were highly expressed in rat brain endothelial cells while the inducible NOS2 showed a low expression level (Fig. 9a). Kainate treatment significantly elevated the NOS2/iNOS gene expression but had no effect on NOS1/nNOS and NOS3/eNOS mRNA expressions as detected by both methods. Dexamethasone attenuated the increased expression of NOS2/iNOS caused by kainate treatment while it did not modify the expression of NOS1/nNOS (Fig. 9). The expression level of NOS3/eNOS mRNA was elevated in the kainate + dexamethasone group measured by RT-PCR, but this result was not confirmed by qPCR.

## Discussion

Excitotoxicity is one of the main pathological pathways in CNS diseases leading to both neuronal injury and BBB dysfunction [5, 42]. Glutamate was shown to increase BBB permeability and reduce the integrity of tight junctions in culture models of the BBB [8, 32, 43]. Ionotropic glutamate receptor agonists NMDA and AMPA were also described to decrease resistance and increase permeability of porcine brain endothelial cells [32]. The same team reported that kainate also induced cell damage, increased DNA fragmentation and cell death in this BBB model, but further direct effects or the presence of kainate receptors were not investigated.

In the present study we demonstrated that kainate decreased cell viability measured by impedance in brain endothelial cells, but not in cultures of brain pericytes or glial cells. While several studies confirm the sensitivity of cultured neurons and oligodendrocytes for kainate toxicity, there are no data in the scientific literature regarding kainate-induced cell death in astrocyte or brain pericyte cultures. The kainate concentration used in our experiment is high, however, both for glutamate and kainate in culture models of the BBB similarly high concentrations were applied [6, 8, 32]. Extracellular glutamate concentrations as high as 50 and 75 µM were measured in the hippocampus of human patients with epilepsy by microdialysis [44] indicating that in pathological conditions very high concentrations of excitotoxic neurotransmitters can be released.

Kainate also increased the permeability of marker molecules fluorescein and albumin across the BBB model and changed the staining pattern of tight junction proteins in brain endothelial cells. These data are in concordance with the barrier opening effect of glutamate in our previous study [8] and with data from other groups [32, 43]. Among the receptors mediating the effect of excitatory mediators several types of the AMPA, NMDA and metabotropic glutamate receptors were described in brain endothelial cells at RNA, protein or functional levels [6–10, 43]. In addition to these receptors, we also found the mRNA expression of GluK1 and GluK4 in isolated brain microvessels and GluK1 in cultured brain endothelial cells. The brain microvessel fractions contain not only brain endothelial cells, but also pericytes and astrocytic endfeet. While brain pericytes are responsive to glutamate [45], there are no data on the presence of kainate receptors in brain pericytes. Astroglia cells express GluK4/KA1 both in vivo [46] and in culture [47].

Although oxidative stress and the production of excess ROS contribute to the damage in excitotoxicity, and kainate was described to increase ROS production in porcine brain endothelial cells [32] in the present experiments kainate had no effect on ROS production in our model. The higher concentration of kainate and the different detection method in the study of Basuroy et al. may explain the dissimilarity [32]. We found that kainate elevated NO production and the band intensity of iNOS mRNA. The level of iNOS is negligible in endothelial cells under physiological conditions, but it is increased in pathologies [48]. In our study the increased mRNA level of iNOS also indicates the damaging effect of kainate. Our observations are in accordance with previous results showing that kainate injection into cerebral cortex increased NOS activity in microvessels in rats [49].

The importance of the BBB as a therapeutic target is increasingly recognized and several anti-inflammatory or antioxidant drugs and novel molecules were identified as BBB protective on culture models [25] and introduced in the therapy of disease like stroke, amyotrophic lateral sclerosis and epilepsy [15–17]. We demonstrated that simvastatin protected brain endothelial cells in the viability and permeability assays against kainate-induced changes and also restored cell morphology. These findings are in agreement with the protective effects of fluvastatin in BBB disruption induced by glutamate in vitro [43]. Simvastatin showed also neuroprotective effect against NMDA-induced excitotoxicity in a culture model [50]. Our data are in concordance with the potential therapeutic use of statins in epilepsy [19], stroke and neurodegenerative diseases [51]. Brain penetration is a key factor in the potential therapeutic efficacy of statins in neurological diseases. Statins can be ligands of solute carriers, like OATPs, and efflux transporters, such as P-glycoprotein, which were identified at the level of the BBB and regulate CNS drug disposition [38, 41]. In a previous study we measured the permeability of rosuvastatin, pravastatin and atorvastatin on our BBB model and found good penetration for these molecules [41].

Edaravone was also protective against kainate induced damage and it reduced ROS levels and kainate-induced NO production in our experiments. These data confirm our previous results on the protective and antioxidant effects of edaravone on cultured brain endothelial cells [23]. Edaravone promoted tight junction formation in vascular endothelial cells [52] and exerted antioxidant effects during NMDA excitotoxicity on brain slices [53]. The protective effect of edaravone was also demonstrated in kainate-induced epilepsy models in rats [24, 54]. All these preclinical data support the BBB protective effects of edaravone which may contribute to its efficacy in the clinical treatment of acute stroke and amyotrophic lateral sclerosis [17].

The barrier tightening effect of dexamethasone was described in several studies using culture models of the BBB [25, 26]. Dexamethasone upregulated claudin-5 and ZO-1 tight junction proteins in damaged brain endothelial cell cultures [55, 56] supporting our data. The observations of the present study confirm data demonstrating the BBB protective effects of dexamethasone obtained in animal models of epilepsy induced by kainate [27] and by pilocarpine [16]. In addition to its barrier protecting effect dexamethasone decreased NO production and the iNOS mRNA expression elevated by kainate in brain endothelial cells. In accordance with our results dexamethasone decreased both BBB permeability and iNOS activity in lipopolysaccharide-treated mice [57]. Although the therapeutic efficacy of dexamethasone in epilepsy is still controversial, it exerted beneficial effects in pediatric drug resistant epileptic patients [16].

## Conclusion

In conclusion, we proved for the first time that the excitotoxin kainate directly damaged cultured brain endothelial cells. Kainate made the immunostaining of junctional proteins discontinuous at the cell border indicating the opening of the BBB model. Kainate increased the permeability of the BBB model for marker molecules, the production of nitric oxide (NO) and the mRNA expression of inducible nitric oxide synthase (NOS2/iNOS) in brain endothelial cells. The presence of kainate receptors was also demonstrated on brain endothelial cells. Simvastatin, edaravone and dexamethasone protected the BBB model against kainate-induced reduced cell viability, increased permeability and morphological changes in cellular junctions. Dexamethasone attenuated the elevated nitric oxide production and decreased the mRNA expression of inducible nitric oxide synthase (NOS2/iNOS) increased by kainate treatment. Our results confirmed the potential clinical usefulness of these drugs to attenuate BBB damage.

## Supplementary information

**Additional file 1.** Detailed protocol, additional figures and tables.

## Data Availability

The dataset used and/or analysed during the current study are available from the corresponding author on reasonable request.

## Abbreviations

AMPA: α-amino-3-hydroxy-5-methyl-4-isoxazolepropionic acid
BBB: blood–brain barrier
BEC: brain endothelial cells
BR: brain
BSA: bovine serum albumin
CNS: central nervous system
DAF-FM: 4-amino-5-methylamino-2′,7′-difluorofluorescein diacetate
DCFDA: chloromethyl-dichloro-dihydro-fluorescein diacetate
DMEM: Dulbecco’s modified Eagle medium
DXM: dexamethasone
EBA: Evans blue-labeled albumin
EDA: edaravone
eNOS/NOS3: endothelial nitric oxide synthase
FBS: fetal bovine serum
H_2_O_2_: hydrogen peroxide
iNOS/NOS2: inducible nitric oxide synthase
KA: kainate
MV: microvessel
NMDA: *N*-methyl-d-aspartate
nNOS/NOS1: neuronal nitric oxide synthase
NO: nitric oxide
P_app_: apparent permeability coefficient
PBS: phosphate buffered saline
PDS: plasma-derived bovine serum
ROS: reactive oxygen species
RT-PCR: reverse transcription polymerase chain reaction
SF: sodium fluorescein
SIM: simvastatin
SNP: sodium nitroprusside
ZO-1: zonula occludens protein-1

## References

[1] Lai TW, Zhang S, Wang YT (2014). Excitotoxicity and stroke: identifying novel targets for neuroprotection. Prog Neurobiol.

[2] Barker-Haliski M, White HS (2015). Glutamatergic mechanisms associated with seizures and epilepsy. Cold Spring Harb Perspect Med..

[3] Schrattenholz A, Soskic V (2006). NMDA receptors are not alone: dynamic regulation of NMDA receptor structure and function by neuregulins and transient cholesterol-rich membrane domains leads to disease-specific nuances of glutamate-signalling. Curr Top Med Chem.

[4] Simeone TA, Sanchez RM, Rho JM (2004). Molecular biology and ontogeny of glutamate receptors in the mammalian central nervous system. J Child Neurol.

[5] Klement W, Blaquiere M, Zub E, deBock F, Boux F, Barbier E (2019). A pericyte-glia scarring develops at the leaky capillaries in the hippocampus during seizure activity. Epilepsia..

[6] Krizbai IA, Deli MA, Pestenácz A, Siklós L, Szabó CA, András I (1998). Expression of glutamate receptors on cultured cerebral endothelial cells. J Neurosci Res.

[7] Parfenova H, Fedinec A, Leffler CW (2003). Ionotropic glutamate receptors in cerebral microvascular endothelium are functionally linked to heme oxygenase. J Cereb Blood Flow Metab.

[8] András IE, Deli MA, Veszelka S, Hayashi K, Hennig B, Toborek M (2007). The NMDA and AMPA/KA receptors are involved in glutamate-induced alterations of occludin expression and phosphorylation in brain endothelial cells. J Cereb Blood Flow Metab.

[9] Scott GS, Bowman SR, Smith T, Flower RJ, Bolton C (2007). Glutamate-stimulated peroxynitrite production in a brain-derived endothelial cell line is dependent on N-methyl-d-aspartate (NMDA) receptor activation. Biochem Pharmacol.

[10] Reijerkerk A, Kooij G, van der Pol SM, Leyen T, Lakeman K, van Het Hof B (2010). The NR1 subunit of NMDA receptor regulates monocyte transmigration through the brain endothelial cell barrier. J Neurochem.

[11] Negri S, Faris P, Pellavio G, Botta L, Orgiu M, Forcaia G (2019). Group 1 metabotropic glutamate receptors trigger glutamate-induced intracellular Ca(^2+^) signals and nitric oxide release in human brain microvascular endothelial cells. Cell Mol Life Sci.

[12] Abbott NJ, Patabendige AA, Dolman DE, Yusof SR, Begley DJ (2010). Structure and function of the blood–brain barrier. Neurobiol Dis.

[13] Sweeney MD, Kisler K, Montagne A, Toga AW, Zlokovic BV (2018). The role of brain vasculature in neurodegenerative disorders. Nat Neurosci.

[14] Krizanac-Bengez L, Mayberg MR, Janigro D (2004). The cerebral vasculature as a therapeutic target for neurological disorders and the role of shear stress in vascular homeostasis and pathophysiology. Neurol Res.

[15] Hachinski V, Einhäupl K, Ganten D, Alladi S, Brayne C, Stephan BCM (2019). Preventing dementia by preventing stroke: the Berlin Manifesto. Alzheimers Dement..

[16] Marchi N, Granata T, Freri E, Ciusani E, Ragona F, Puvenna V (2011). Efficacy of anti-inflammatory therapy in a model of acute seizures and in a population of pediatric drug resistant epileptics. PLoS ONE.

[17] Watanabe K, Tanaka M, Yuki S, Hirai M, Yamamoto Y (2018). How is edaravone effective against acute ischemic stroke and amyotrophic lateral sclerosis?. J Clin Biochem Nutr..

[18] Ramirez C, Tercero I, Pineda A, Burgos JS (2011). Simvastatin is the statin that most efficiently protects against kainate-induced excitotoxicity and memory impairment. J Alzheimers Dis..

[19] Banach M, Czuczwar SJ, Borowicz KK (2014). Statins—are they anticonvulsant?. Pharmacol Rep..

[20] Oesterle A, Laufs U, Liao JK (2017). Pleiotropic effects of statins on the cardiovascular system. Circ Res.

[21] Sierra S, Ramos MC, Molina P, Esteo C, Vázquez JA, Burgos JS (2011). Statins as neuroprotectants: a comparative in vitro study of lipophilicity, blood–brain-barrier penetration, lowering of brain cholesterol, and decrease of neuron cell death. J Alzheimers Dis..

[22] Nagaraja TN, Knight RA, Croxen RL, Konda KP, Fenstermacher JD (2006). Acute neurovascular unit protection by simvastatin in transient cerebral ischemia. Neurol Res.

[23] Tóth AE, Walter FR, Bocsik A, Sántha P, Veszelka S, Nagy L (2014). Edaravone protects against methylglyoxal-induced barrier damage in human brain endothelial cells. PLoS ONE.

[24] Nomura S, Shimakawa S, Miyamoto R, Fukui M, Tamai H (2014). 3-Methyl-1-phenyl-2-pyrazolin-5-one or N-acetylcysteine prevents hippocampal mossy fiber sprouting and rectifies subsequent convulsive susceptibility in a rat model of kainic acid-induced seizure ceased by pentobarbital. Brain Res.

[25] Deli MA, Ábrahám CS, Kataoka Y, Niwa M (2005). Permeability studies on in vitro blood–brain barrier models: physiology, pathology, and pharmacology. Cell Mol Neurobiol.

[26] Förster C, Kahles T, Kietz S, Drenckhahn D (2007). Dexamethasone induces the expression of metalloproteinase inhibitor TIMP-1 in the murine cerebral vascular endothelial cell line cEND. J Physiol.

[27] Sztriha L, Joó F, Szerdahelyi P, Eck E, Koltai M (1986). Effects of dexamethasone on brain edema induced by kainic acid seizures. Neuroscience.

[28] Walter FR, Valkai S, Kincses A, Petneházi A, Czeller T, Veszelka S (2016). A versatile lab-on-a-chip tool for modeling biological barriers. Sens Actuators B: Chem..

[29] Harazin A, Bocsik A, Barna L, Kincses A, Váradi J, Fenyvesi F (2018). Protection of cultured brain endothelial cells from cytokine-induced damage by α-melanocyte stimulating hormone. PeerJ..

[30] Perrière N, Demeuse P, Garcia E, Regina A, Debray M, Andreux JP (2005). Puromycin-based purification of rat brain capillary endothelial cell cultures Effect on the expression of blood–brain barrier-specific properties. J Neurochem.

[31] Nakagawa S, Deli MA, Kawaguchi H, Shimizudani T, Shimono T, Kittel A (2009). A new blood–brain barrier model using primary rat brain endothelial cells, pericytes and astrocytes. Neurochem Int.

[32] Basuroy S, Leffler CW, Parfenova H (2013). CORM-A1 prevents blood–brain barrier dysfunction caused by ionotropic glutamate receptor-mediated endothelial oxidative stress and apoptosis. Am J Physiol Cell Physiol.

[33] Kiss L, Walter FR, Bocsik A, Veszelka S, Ozsvári B, Puskás LG (2013). Kinetic analysis of the toxicity of pharmaceutical excipients Cremophor EL and RH40 on endothelial and epithelial cells. J Pharm Sci.

[34] Bernard A, Ferhat L, Dessi F, Charton G, Represa A, Ben-Ari Y (1999). Q/R editing of the rat GluR5 and GluR6 kainate receptors in vivo and in vitro: evidence for independent developmental, pathological and cellular regulation. Eur J Neurosci.

[35] Hinoi E, Yoneda Y (2001). Expression of GluR6/7 subunits of kainate receptors in rat adenohypophysis. Neurochem Int.

[36] Maric D, Liu QY, Grant GM, Andreadis JD, Hu Q, Chang YH (2000). Functional ionotropic glutamate receptors emerge during terminal cell division and early neuronal differentiation of rat neuroepithelial cells. J Neurosci Res.

[37] Yao SY, Ljunggren-Rose A, Chandramohan N, Whetsell WO, Sriram S (2010). In vitro and in vivo induction and activation of nNOS by LPS in oligodendrocytes. J Neuroimmunol.

[38] Campos-Bedolla P, Walter FR, Veszelka S, Deli MA (2014). Role of the blood–brain barrier in the nutrition of the central nervous system. Arch Med Res.

[39] Veszelka S, Tóth AE, Walter FR, Datki Z, Mózes E, Fülöp L (2013). Docosahexaenoic acid reduces amyloid-β induced toxicity in cells of the neurovascular unit. J Alzheimers Dis..

[40] Lénárt N, Walter FR, Bocsik A, Sántha P, Tóth ME, Harazin A (2015). Cultured cells of the blood–brain barrier from apolipoprotein B-100 transgenic mice: effects of oxidized low-density lipoprotein treatment. Fluids Barriers CNS..

[41] Veszelka S, Tóth A, Walter FR, Tóth AE, Gróf I, Mészáros M (2018). Comparison of a rat primary cell-based blood–brain barrier model with epithelial and brain endothelial cell lines: gene expression and drug transport. Front Mol Neurosci..

[42] Friedman A, Heinemann U. Role of blood–brain barrier dysfunction in Epileptogenesis. In: Noebels JL, Avoli M, Rogawski MA, Olsen RW, Delgado-Escueta AV, editors. Jasper’s basic mechanisms of the Epilepsies 4th edition. Bethesda (MD): National Center for Biotechnology Information (US); 2012. http://www.ncbi.nlm.nih.gov/books/NBK98210/. 22787606

[43] Kuhlmann CR, Gerigk M, Bender B, Closhen D, Lessmann V, Luhmann HJ (2008). Fluvastatin prevents glutamate-induced blood–brain-barrier disruption in vitro. Life Sci.

[44] During MJ, Spencer DD (1993). Extracellular hippocampal glutamate and spontaneous seizure in the conscious human brain. Lancet.

[45] Hall CN, Reynell C, Gesslein B, Hamilton NB, Mishra A, Sutherland BA (2014). Capillary pericytes regulate cerebral blood flow in health and disease. Nature.

[46] Vargas JR, Takahashi DK, Thomson KE, Wilcox KS (2013). The expression of kainate receptor subunits in hippocampal astrocytes after experimentally induced status epilepticus. J Neuropathol Exp Neurol.

[47] Cauley K, Kukekov V, Young D (1997). Kainate/AMPA receptors expressed on human fetal astrocytes in long-term culture. J Neurosci Res.

[48] Naseem KM (2005). The role of nitric oxide in cardiovascular diseases. Mol Aspects Med.

[49] Lei DL, Yang DL, Liu HM (1996). Local injection of kainic acid causes widespread degeneration of NADPH-d neurons and induction of NADPH-d in neurons, endothelial cells and reactive astrocytes. Brain Res.

[50] Zacco A, Togo J, Spence K, Ellis A, Lloyd D, Furlong S (2003). 3-hydroxy-3-methylglutaryl coenzyme A reductase inhibitors protect cortical neurons from excitotoxicity. J Neurosci.

[51] Malfitano AM, Marasco G, Proto MC, Laezza C, Gazzerro P, Bifulco M (2014). Statins in neurological disorders: an overview and update. Pharmacol Res.

[52] Onodera H, Arito M, Sato T, Ito H, Hashimoto T, Tanaka Y (2013). Novel effects of edaravone on human brain microvascular endothelial cells revealed by a proteomic approach. Brain Res.

[53] Nakano-Okuda Y, Hasegawa K, Hirai K, Kanai-Ochiai R, Morimoto M, Sugimoto T (2006). Effects of edaravone on N-methyl-d-aspartate (NMDA)-mediated cytochrome c release and apoptosis in neonatal rat cerebrocortical slices. Int J Dev Neurosci.

[54] Miyamoto R, Shimakawa S, Suzuki S, Ogihara T, Tamai H (2008). Edaravone prevents kainic acid-induced neuronal death. Brain Res.

[55] Blecharz KG, Haghikia A, Stasiolek M, Kruse N, Drenckhahn D, Gold R (2010). Glucocorticoid effects on endothelial barrier function in the murine brain endothelial cell line cEND incubated with sera from patients with multiple sclerosis. Mult Scler..

[56] Hue CD, Cho FS, Cao S, Dale Bass CR, Meaney DF, Morrison B (2015). Dexamethasone potentiates in vitro blood–brain barrier recovery after primary blast injury by glucocorticoid receptor-mediated upregulation of ZO-1 tight junction protein. J Cereb Blood Flow Metab.

[57] Minami T, Okazaki J, Kawabata A, Kawaki H, Okazaki Y, Tohno Y (1998). Roles of nitric oxide and prostaglandins in the increased permeability of the blood–brain barrier caused by lipopolysaccharide. Environ Toxicol Pharmacol.

