# Supplementary material for: Simvastatin, edaravone and dexamethasone protect against kainate-induced brain endothelial cell damage
Source: Fluids Barriers CNS. 2020 Feb 10;17:5. doi: 10.1186/s12987-019-0166-1 (PMC7008534; doi:10.1186/s12987-019-0166-1)
Supplement: Supplementary file 1 — Additional file 1. Detailed protocol, additional figures and tables. [file 12987_2019_166_MOESM1_ESM.pdf]

Additional file 1: Detailed protocol, additional figures and tables.

Simvastatin, edaravone and dexamethasone protect against kainate-induced brain endothelial cell damage

Lilla Barna, Fruzsina R. Walter, András Harazin, Alexandra Bocsik, András Kincses, Vilmos Tubak, Katalin Jósvay, Ágnes Zvara, Patricia Campos-Bedolla, Mária A Deli

---

Detailed protocol

Primary rat brain endothelial cells were isolated from three-week-old male and female Wistar rats (Harlan Laboratories, USA), as it is described in our previous papers [Walter et al., 2016; Harazin et al., 2018]. Animals were sacrificed with CO_2 then forebrains were collected in phosphate buffered saline (PBS) on ice. After removing meninges, the tissue was cut into 1 mm^3 pieces and enzymatically digested by 1 mg/ml collagenase type II (Roche, Switzerland), and 15 µg/ml deoxyribonuclease type I (Roche, Switzerland) in Dulbecco's modified Eagle medium/Nutrient Mixture F-12 (DMEM/F12, Gibco, Life Technologies, USA) for 55 min at 37 °C. After the digestion the cells were mixed with 20% bovine serum albumin (BSA)-DMEM and centrifuged three times (1,000 × *g*, 20 min) to separate the brain microvessel fraction from the myelin rich brain tissue fraction. After each centrifugation step the cell pellets were collected and pooled. The collected microvessels were further digested by 1 mg/ml collagenase-dispase (Roche, Switzerland) in DMEM/F12 containing 15 µg/ml deoxyribonuclease type I (Roche, Switzerland) for 35 min at 37 °C. Brain microvascular endothelial cell clusters were separated on a 33 % Percoll gradient (1,000 × *g*, 10 min) then collected and washed twice in DMEM/F12. Cells were seeded onto collagen type IV and fibronectin coated (100 µg/ml each) Petri dishes (100 mm; Corning, USA). Cells were maintained in DMEM-F12 supplemented with 15 % plasma-derived bovine serum (PDS; First Link, UK), 5 µg/ml insulin, 5 µg/ml transferrin, 5 ng/ml sodium selenite (Pan Biotech, Germany), 10 mM Hepes, 1 ng/ml basic fibroblast growth factor, 100 µg/ml heparin and 50 µg/ml gentamycin. During the first three days of culture the capillary endothelial cells were kept in culture medium containing 3 µg/ml puromycin to eliminate P-glycoprotein negative cell types [Perrière et al., 2005]. Cells were used at the first passage for experiments.

Primary rat brain pericytes were isolated by the same protocol, except that puromycin treatment was not applied. After isolation pericytes were seeded onto collagen type IV coated

(100 µg/ml) Petri dishes (60 mm; VWR International, USA) and cultured in DMEM medium (Gibco, Life Technologies, USA) containing 10 % fetal bovine serum (FBS, Pan Biotech, Germany) and gentamycin (50 µg/ml). Cells were used at the third passage for experiments.

Primary rat astrocytes were obtained from 1-day-old Wistar rats. Meninges were removed then the brain tissue was mechanically dissociated with a long needle connected to a syringe. The homogenate was filtered through a nylon mesh (40 µm, Millipore, USA) to remove larger vessels and tissue debris. Cell clusters were plated onto uncoated 75 cm^2 flasks (TPP, Switzerland) and cultured in DMEM supplemented with 10 % FBS and gentamycin (50 µg/ml) until they reached 90 % confluency. For the BBB co-culture model cells were cultured for 2 weeks (first passage) before use in appropriate collagen type IV (100 µg/ml) coated 12-well plates (Corning, USA) at a cell number of 5×10^4 cells/well. Confluent glia cultures included 90 % GFAP immunopositive astroglia and 10 % CD11b immunopositive microglia.

Brain endothelial cells were co-cultured with brain pericytes and astrocytes to induce the BBB characteristics [Nakagawa et al., 2009]. First pericytes (1.5×10^4 cells/cm^2) were passaged to the bottom side of the 12-well format culture inserts (Transwell clear, polyester membrane, 0.4 µm pore size, 1.12 cm^2 surface; Corning, USA) coated with collagen type IV (100 µg/ml), then brain endothelial cells (7.5×10^4 cells/cm^2) were added to the upper side coated with collagen type IV and fibronectin (100 µg/ml each). The inserts were placed into 12-well plates containing confluent astrocyte layers. Endothelial culture medium was added to both compartments. Cells were co-cultured for four days before experiments [Perrière et al., 2005].

Harazin A, Bocsik A, Barna L, Kincses A, Váradi J, Fenyvesi F, et al. Protection of cultured brain endothelial cells from cytokine-induced damage by α-melanocyte stimulating hormone. PeerJ. 2018;6:e4774. https://doi.org/10.7717/peerj.4774.

Nakagawa S, Deli MA, Kawaguchi H, Shimizudani T, Shimono T, Kittel A, et al. A new blood-brain barrier model using primary rat brain endothelial cells, pericytes and astrocytes. Neurochem Int. 2009;54(3-4):253-63. https://doi.org/10.1016/j.neuint.2008.12.002.

Perrière N, Demeuse P, Garcia E, Regina A, Debray M, Andreux JP, et al. Puromycin-based purification of rat brain capillary endothelial cell cultures. Effect on the expression of blood-brain barrier-specific properties. J Neurochem. 2005;93(2):279-89.

Walter FR, Valkai S, Kincses A, Petneházi A, Czeller T, Veszelka S, et al. A versatile lab-on-a-chip tool for modeling biological barriers, Sensors and Actuators B: Chemical. 2016;222:1209-19. https://doi.org/10.1016/j.snb.2015.07.110.

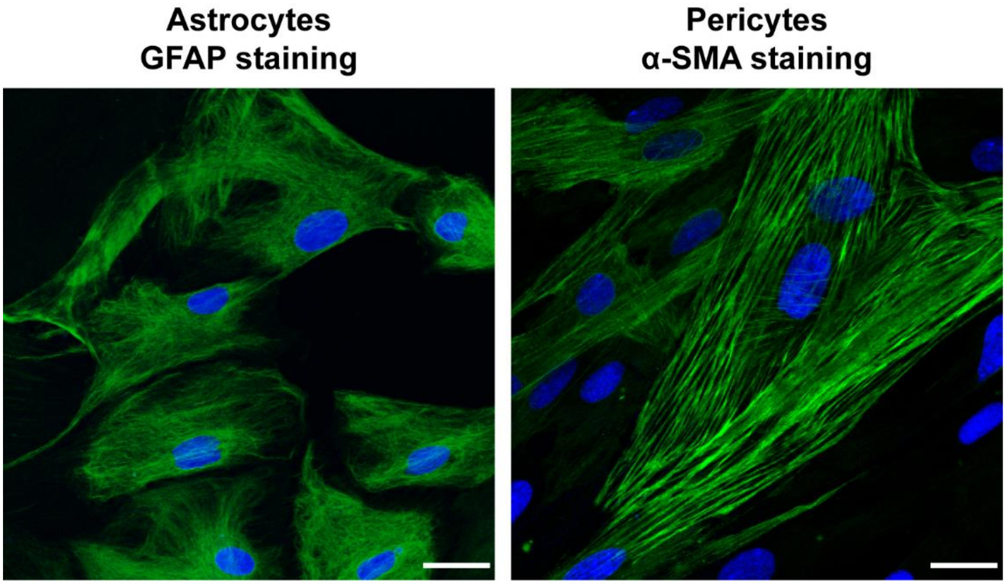

Figure S1. Characterization of primary rat astrocytes and pericytes. Cell morphology was characterized by glial fibrillary acidic protein (GFAP, astroglia) and α-smooth muscle actin (α-SMA, pericyte) immunostaining and visualized by confocal microscopy (Leica TCS SP5, confocal laser scanning microscope; Leica Microsystems, Germany). Cells were fixed with cold acetone-methanol solution (1:1) for 10 min, washed with phosphate buffered saline (PBS) and non-specific binding sites were blocked with 3% BSA-PBS for 1 h at room temperature. Incubation with mouse anti-α-SMA (Dako, USA) and mouse anti-GFAP primary antibodies lasted overnight at 4 °C. Cells were incubated with anti-rabbit secondary antibody labeled with Cy3 or anti-mouse secondary antibody labeled with Alexa Fluor 488 (Life Technologies, USA) and H33343 dye to stain nuclei for 1 h at room temperature. Between incubations cells were washed three times with PBS. Scale bar = 25 μm.

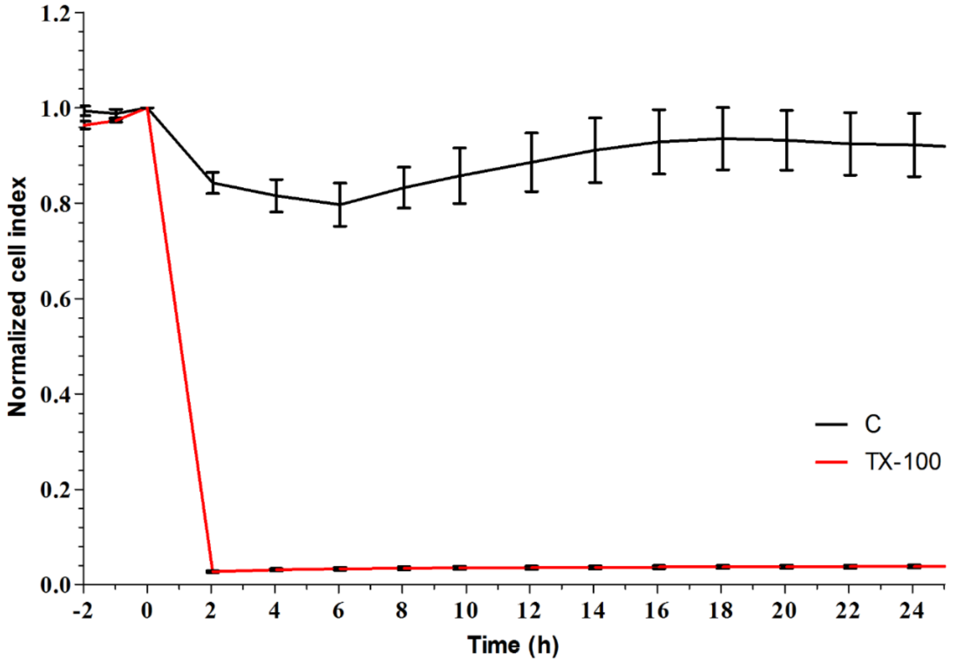

Figure S2. Effect of Triton X-100 (TX-100) on cell viability measured by impedance kinetics. Triton X-100 detergent was used at 1% concentration as a reference compound to cause cell death. The impedance of the control group (C) remained stable during the 24-h period.

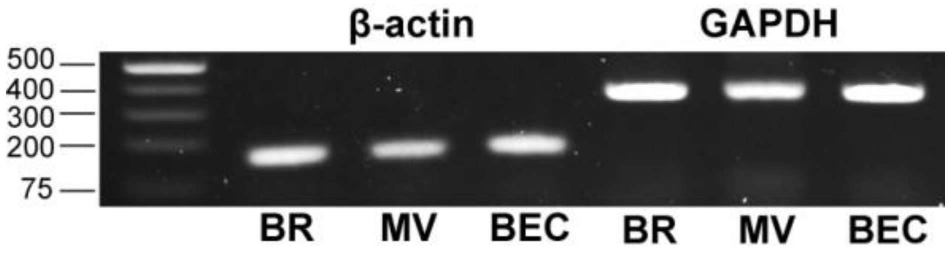

Figure S3. Expression of housekeeping genes (β-actin and glyceraldehyde 3-phosphate dehydrogenase – GAPDH) in rat brain cortex (BR), isolated rat brain microvessels (MV) and primary rat brain endothelial cells (BEC). The predicted length of the products is shown in Table S2. Fragments were visualized on 2% agarose gel. M: 1 kb Plus DNA ladder.

Table S1. Origin and catalogue number of reagents used in this study

Product name	Company	Country	Catalogue number
collagenase type II	Roche	Switzerland	C6885
deoxyribonuclease type I	Roche	Switzerland	D4513
Dulbecco's modified Eagle medium (DMEM)	Gibco, Life Technologies	USA	21885025
Dulbecco's Modified Eagle Medium/Nutrient Mixture F-12 (DMEM/F12)	Gibco, Life Technologies	USA	31331028
bovine serum albumin (BSA)	Sigma, Merck	Germany	A7906
collagenase-dispase	Roche	Germany	11097 113001
Percoll	Sigma, Merck	Germany	P4937
collagen type IV	Sigma, Merck	Germany	C5533
fibronectin	Sigma, Merck	Germany	F1141
plasma-derived bovine serum (PDS)	First Link	UK	60-00-850
insulin	Pan Biotech	Germany	P07-03110
transferrin			
sodium selenite			
Hepes	Sigma, Merck	Germany	H4034
basic fibroblast growth factor	Sigma, Merck	Switzerland	F0291
heparin	Sigma, Merck	Germany	H3149
gentamycin	Sigma, Merck	Germany	G1397
puromycin	Sigma, Merck	Germany	P7255
fetal bovine serum (FBS)	Pan Biotech	Germany	P30-3702
kainic acid	Sigma, Merck	Germany	K-0250
simvastatin	Sigma, Merck	Germany	S-6196
edaravone	Sigma, Merck	Germany	M70800
dexamethasone	Sigma, Merck	Germany	D2915
hydrogen peroxide (H2O2)	Molar Chemicals	Hungary	03650
sodium nitroprusside (SNP)	Sigma, Merck	Germany	71778
collagen type I	isolated in our laboratory	-	-
TriFast reagent	VWR International	USA	30-2010
Maxima First Strand cDNA Synthesis Kit	Thermo Fisher	USA	K1671
FIREPol DNA Polymerase	Solis BioDyne	Estonia	01-01-01000

agarose	VWR International	USA	443666A
Evans blue	Sigma-Aldrich	USA	E2129
sodium fluorescein	Sigma-Aldrich	USA	F6377
chloromethyl-dichloro-dihydro-fluorescein diacetate (DCFDA)	Molecular Probes, Life Technologies	USA	C6827
4-amino-5-methylamino-2’,7’-difluorofluorescein diacetate (DAF-FM)	Molecular Probes, Life Technologies	USA	D23844
pluronic acid	Molecular Probes, Life Technologies	USA	P6867
paraformaldehyde	Sigma-Aldrich	USA	441244
Triton-X100	AppliChem	Germany	A4975
rabbit anti-claudin-5 polyclonal antibody	Sigma-Aldrich	USA	SAB4502981
rabbit anti-ZO-1 polyclonal antibody	Invitrogen	USA	61-7300
anti-rabbit secondary antibody conjugated with Cy3	Sigma-Aldrich	USA	C2306
H33342 nucleus stain	Calbiochem	USA	382065
Fluoromount-G	Southern Biotech	USA	0100-01

Table S2. Rat-specific primers used for RT-PCR.

Primer name		Sequence	Tm (°C)	Amplicon length (bp)	Gene ID
**GluK1/ GluR5**	fw	GGTATAACCCCCACCCATGCAACC	58	314	NM_017241.2
	rv	GAAGGTCATCGTCGAGCCATCTCTG			
**GluK2/ GluR6**	fw	GGTATAACCCACACCCTTGCAACC	56	452	NM_019309.2
	rv	TGACTCCATTAAGAAAGCATAATCCGA			
**GluK3/ GluR7**	fw	CTTCTTCAAGAAATCCAAGATCTCCACC	57	483	NM_181373.3
	rv	TGCTCCCGTTCCGCTGTCTTGC			
**GluK4/ KA-1**	fw	TACTCTCTGGGCAACAGCCT	56	289	NM_012572.1
	rv	AGGTCTGGTAGCGGGAATTT			
**GluK5/ KA-2**	fw	TCGCCCGTGTCCTCAACTCA	55	398	NM_031508.2
	rv	CACCGACACCTCCTCAGACTCCG			
**nNOS/ NOS1**	fw	TCTACGCCACAGAGACAGGCAAAT	51	92	NM_052799
	rv	CATGGACATTGCCTTGGCATCGAA			
**iNOS/ NOS2**	fw	AGCATCCCAAGTACGAGT	48	140	NM_012611
	rv	AATCTCGGTGCCCATGTA			
**eNOS/ NOS3**	fw	CGGAGAATGGAGAGAGCTTT	50	113	NM_021838
**β-actin**	fw	TACTCTGTGTGGATTGGTGGC	52	150	NM_031144.3
	rv	GGTGTAAAACGCAGCTCAGTAA			
**GAPDH**	fw	GGTCATCAACGGGAAACCC	55	356	NM_017008.4
	rv	TCTGAGTGGCAGTGATGGCA			
